# Patient-relevant health outcomes for von Willebrand disease, platelet function disorders, and rare bleeding disorders: a Delphi study

**DOI:** 10.1016/j.rpth.2023.102201

**Published:** 2023-09-14

**Authors:** Evelien S. van Hoorn, Hester F. Lingsma, Marjon H. Cnossen, Samantha C. Gouw

**Affiliations:** 1 Department of Public Health, Erasmus University Medical Center Rotterdam, Rotterdam, The Netherlands; 2 Department of Pediatric Hematology and Oncology, Sophia Children’s Hospital, Erasmus University Medical Center, Rotterdam, The Netherlands; 3 Department of Pediatric Hematology, Amsterdam University Medical Centers location University of Amsterdam, Amsterdam, The Netherlands

**Keywords:** caregivers, consensus, menstruation, value-based health care, von Willebrand diseases

## Abstract

**Background:**

To assess patient value, it is essential to regularly measure health outcomes that matter to patients. It is currently unknown which health outcomes are important for patients with autosomal inherited bleeding disorders.

**Objectives:**

This study aimed to assess which health outcomes are important for patients with autosomal inherited bleeding disorders, consisting of von Willebrand disease, platelet function disorders, and rare bleeding disorders, as seen from the patients’, caregivers’, and healthcare professionals’ perspectives.

**Methods:**

Two panels, one consisting of patients and caregivers, and one consisting of healthcare professionals participated in a Delphi process. A list of 146 health outcomes was identified from the literature. During 3 rounds, both panels rated the importance of health outcomes on a 5-point Likert scale. A health outcome was considered important by a panel if it received a median score of 5 with an IQR of ≤1.

**Results:**

In total, 13 patients, 10 caregivers, and 19 healthcare professionals participated in the Delphi study. Both panels reached consensus on the importance of health outcomes related to bleeding episodes, life-threatening complications, and the intensity and impact of menstruation. Patients and caregivers additionally reached consensus on the importance of health outcomes related to menstruation and the impact of the bleeding disorder on their daily lives. Healthcare professionals reached consensus on the importance of health outcomes related to treatment, joint health, and pain.

**Conclusion:**

In this study, health outcomes were identified that should be considered when implementing value-based health care in the care of patients with autosomal inherited bleeding disorders.

## Essentials

• To improve patient experience, it is essential to measure health outcomes that matter to patients.
• We identified health outcomes important for patients with inherited bleeding disorders.
• Patients, caregivers, and healthcare professionals prioritized different health outcomes.
• The identified health outcomes should be considered when implementing value-based health care.

## 1 Introduction

The group of patients with an autosomal inherited bleeding disorder predominantly consists of patients with von Willebrand disease, followed by patients with hereditary rare bleeding disorders or inherited platelet function disorders. These bleeding disorders are caused by different defects in the hemostatic process. These defects lead to heterogeneous bleeding phenotypes which range from mild bleeding, such as easy bruising and epistaxis, to severe or even life-threatening bleeding manifestations as gastrointestinal or intracranial bleeding [[1], [2], [3]]. Additionally, women may experience menorrhagia and postpartum hemorrhage [4,5]. This heterogeneity in clinical presentation often complicates the diagnosis and treatment of autosomal inherited bleeding disorders and calls for personalized treatment strategies.

In recent years, the trend to move healthcare services toward value-based organizations has emphasized the importance of creating value for patients [[6], [7], [8]]. Value-based health care was introduced by Porter and Teisberg [9] in 2006 with the aim of improving patient value, which is defined as health outcomes that matter to patients divided by the cost of achieving those outcomes [6]. By focusing on improving outcomes that matter to patients, healthcare services are in theory able to 1) align their care to drive improvement in the health outcomes that matter most to both patients and healthcare professionals, 2) improve how patients experience their health, and 3) reduce the complexity and progression of disease that drive the need for more care [10]. The systematic measurement of outcomes that matter to patients in clinical practice is therefore indispensable to improving patient value [6,10].

Previous research has focused on determining which health outcomes are important for many different patient populations, including persons with hemophilia [[11], [12], [13]]. It is uncertain whether these identified health outcomes that are important for persons with hemophilia are transferable to patients with autosomal inherited bleeding disorders. Whereas the hemophilia population largely consists of men, a large proportion of patients with autosomal inherited bleeding disorders comprises females, who experience sex-specific health outcomes, such as menorrhagia and pregnancy complications [14,15]. Therefore, this study aimed to assess which health outcomes are important specifically for patients with autosomal inherited bleeding disorders, consisting of von Willebrand disease, platelet function disorders, and rare bleeding disorders, from the patients’, caregivers’, and healthcare professionals’ perspectives.

## 2 Method

### 2.1 Study design

The Delphi methodology was used to establish consensus on which health outcomes are important for patients with autosomal inherited bleeding disorders. The Delphi method is a consensus-based, iterative process that uses successive anonymous surveys to gather information from a selected panel of experts on a specific topic [16,17].

### 2.2 Participant selection and recruitment

tTwo Delphi panels were assembled consisting of Dutch-speaking patients aged ≥18 years with an autosomal inherited bleeding disorder and caregivers of pediatric patients with an autosomal inherited bleeding disorder, and healthcare professionals from various disciplines currently working in the field of bleeding disorders. Healthcare professionals were recruited through professional contacts of the researchers at the 6 hemophilia treatment centers in The Netherlands, the *Dutch Hemophilia Physicians Society*, and the *Dutch Hemophilia Nurses Society*. Patients and caregivers were recruited through the *Dutch Hemophilia Patient Society*. In addition, caregivers of patients currently receiving treatment at the pediatric hematology outpatient clinic of the *Erasmus MC Sophia’s Children Hospital*, in The Netherlands were actively recruited to participate in this study. All contacted individuals or organizations received an email containing a short study description, a description of what is expected of the participant, and a participant information letter. Using a snowballing technique, all contacted individuals were asked to spread the invitation among their network.

Individuals willing to participate in the study could use a link provided in the email to fill in a digital informed consent form. Once the informed consent was given, the participants received a personal login code to complete the Delphi surveys in the web-based Delphi platform Welphi [18]. Using Welphi, follow-up emails were sent 2 weeks and 5 days before the closing of each Delphi round to encourage participation. The study was reviewed and determined to be exempt by the *Medical Ethical Research Committee of the Erasmus MC*, *University Medical Center Rotterdam*, Rotterdam, The Netherlands.

### 2.3 Delphi process

All 3 Delphi surveys included an introduction page that paraphrased the survey’s intent, the study objectives, and an explanation of the Delphi method. Based on the participant characteristics (ie, a patient, caregiver, or healthcare professional), the participant was directed to the specific survey questions.

Before the first Delphi round, a long list of health outcomes was developed based on the long-list used by the CoreHEM and Haemovalue initiatives on health outcomes for persons with hemophilia [12,19]. These long-lists were enriched with the findings of a systematic review on patient-reported outcomes in patients with autosomal inherited bleeding disorders [14]. A plain language definition was provided for each health outcome.

The full Delphi process consisted of 3 rounds. During the first round, both panels were asked to rate each health outcome on the long-list using a 5-point Likert scale, ranging from 1 (not important) to 5 (very important) in terms of each outcome’s importance for patients with autosomal inherited bleeding disorders. To capture uncertainty, an “I do not know” option was also available. Participants were able to motivate and add explanatory notes on their decisions, to provide recommendations on the definition, and to add any additional health outcomes that they deemed important.

All participants who filled in the informed consent were invited to complete the second and third round survey, regardless of their participation in previous rounds. In these rounds, both panels’ collective rating of each outcome’s importance was presented alongside the individual’s rating of the previous round. Participants were able to view the collective round rating as the percentage of participants or the absolute number of participants that rated an outcome on each of the options of the 5-point Likert scale. Additionally, any comments made by the participants in the previous rounds were displayed. This enabled participants to consider the opinions of other participants while deciding on the importance of the various health outcomes.

Health outcomes were excluded from the second round if there was consensus on importance or consensus on unimportance during the first round, unless meaningful adjustments were suggested to the definition. Consensus on importance was defined as a median score of 5 and an IQR of ≤1. In other words, to achieve consensus on importance at least 75% of the panel had to score the health outcome with a 5. The other 25% of the panel had to score the health outcome with a 4. Consensus of unimportance was defined as a median score <5 and an IQR of ≤1. To achieve consensus on unimportance no more than 25% of the panel had to score the health outcome with a 5. The second round therefore consisted primarily of health outcomes on which no consensus was reached. In this second round, participants were again asked to motivate their decisions, to provide recommendations on the definitions, and to add any missing health outcomes that they deemed important.

In the third and final round, health outcomes on which consensus on importance was achieved during the first or second round, and on which no consensus was achieved in the second round were presented to the participants for rating. In this final round, participants were not able to provide recommendations for the definition or add health outcomes.

### 2.4 Statistical analysis

Descriptive statistics were used to describe both panels’ demographics and to determine which health outcomes were selected for the next round. The “I do not know” option was coded as missing. Missing data were excluded from the analysis per health outcome.

Statistical analysis was performed using R statistical language.

## 3 Results

### 3.1 Delphi panel

The Delphi rounds were conducted between February 1, 2021 and February 28, 2022. During the first Delphi round, the Dutch Hemophilia Physicians Society, the Dutch Hemophilia Nurses Society, the Dutch Hemophilia Patient Society, 17 healthcare professionals, and 256 caregivers of patients received an invitation to participate in this study. The informed consent form was signed by 49 participants. Forty-two participants, consisting of 19 healthcare professionals (45%), 13 patients (31%), and 10 caregivers (24%), participated in the first Delphi round (Tables 1 and 2). Most of the participants were female (*n* = 33) and the median age was 41 years (IQR, 35-48 years). The response rate in round 3 was 66.6% (*n* = 32; Supplementary Table S1).

**Table 1:** Demographics of the patient and caregiver panel during the first Delphi round

Characteristics	Patients *n* =13	Caregivers *n* = 10
Sex^a^		
Male	3 (23%)	- (0%)
Female	10 (77%)	7 (100%)
Age, y^a^		
Median	41	38
IQR	36-45	34-43
Educational level^a^		
Lower secondary education	2 (15%)	- (0%)
Higher secondary education	6 (46%)	4 (57%)
Bachelor’s or equivalent education	4 (31%)	1 (14%)
Master, doctoral or equivalent education	1 (8%)	2 (29%)
Type of bleeding disorder^a^^,^^b^		
Von Willebrand disease	10 (77%)	5 (56%)
Fibrinogen deficiency	1 (8%)	- (0%)
FVII deficiency	- (0%)	1 (11%)
FXI deficiency	- (0%)	1 (11%)
FXIII deficiency	- (0%)	1 (11%)
Inherited platelet function disorder	2 (15%)	1 (11%)
Self-reported severity of the bleeding disorder^a^^,^^b^		
Severe	3 (23%)	1 (11%)
Moderate	3 (23%)	2 (2250)
Mild	6 (46%)	6 (67%)
Unknown	1 (8%)	- (0%)
Use of medication^a^^,^^b^		
Yes	10 (77%)	2 (25%)
No	3 (23%)	6 (75%)
Currently receiving treatment at a hemophilia treatment center^a^^,^^b^		
Yes	9 (69%)	3 (43%)
No	3 (23%)	4 (57%)
No treatment necessary	1 (8%)	- (0%)

Data on ethnicity were not collected because it is not allowed under Dutch law.

F, factor; IQR, interquartile range.

a These questions contained missing values for the caregivers.

b Caregivers answered these questions about their child.

**Table 2:** Demographics of the healthcare professionals panel during the first Delphi round.

Characteristics	Healthcare professionals *n* = 19
Sex^a^	
Male	2 (11%)
Female	16 (89%)
Age, y^a^	
Median	48
IQR	40 - 57
Educational level^a^	
Lower secondary education	- (0%)
Higher secondary education	1 (6%)
Bachelor’s or equivalent education	4 (22%)
Master, doctoral, or equivalent education	13 (72%)
Profession	
Hematologist	5 (26%)
Pediatric hematologist	3 (16%)
Nurse consultant	2 (11%)
Nurse practitioner	6 (32%)
Physical therapist	2 (11%)
Other	1 (5%)
Patient population cared for^a^	
Children	6 (33%)
Adults	8 (44%)
Both children and adults	4 (22%)
Years of experience	
Median	20
IQR	8,5 - 27
Currently working at a hemophilia treatment center	
Yes	19 (100%)
No	- (0%)

Data on ethnicity were not collected because it is not allowed under Dutch law.

IQR, interquartile range.

a These questions contained missing values.

### 3.2 Health outcome selection

#### 3.2.1 Round 1

In the first Delphi round, both panels voted on the importance of 146 health outcomes (Figure). The patient and caregiver panels achieved consensus on the importance of 20 health outcomes. The healthcare professionals panel achieved consensus on the importance of 41 health outcomes. Respectively, 102 and 81 health outcomes were excluded due to consensus on unimportance by the patients and caregivers and healthcare professionals panels. During the first round, the panels proposed alterations in the definition for the health outcome vitality and suggested the addition of 5 new health outcomes: 1) concerns about heredity, 2) concerns about pregnancy/miscarriage/giving birth, 3) joint imaging (magnetic resonance imaging or X-ray), 4) visualization of joint bleeds, and 5) increase in joint bleeds (Supplementary Table S2).

**Figure:**
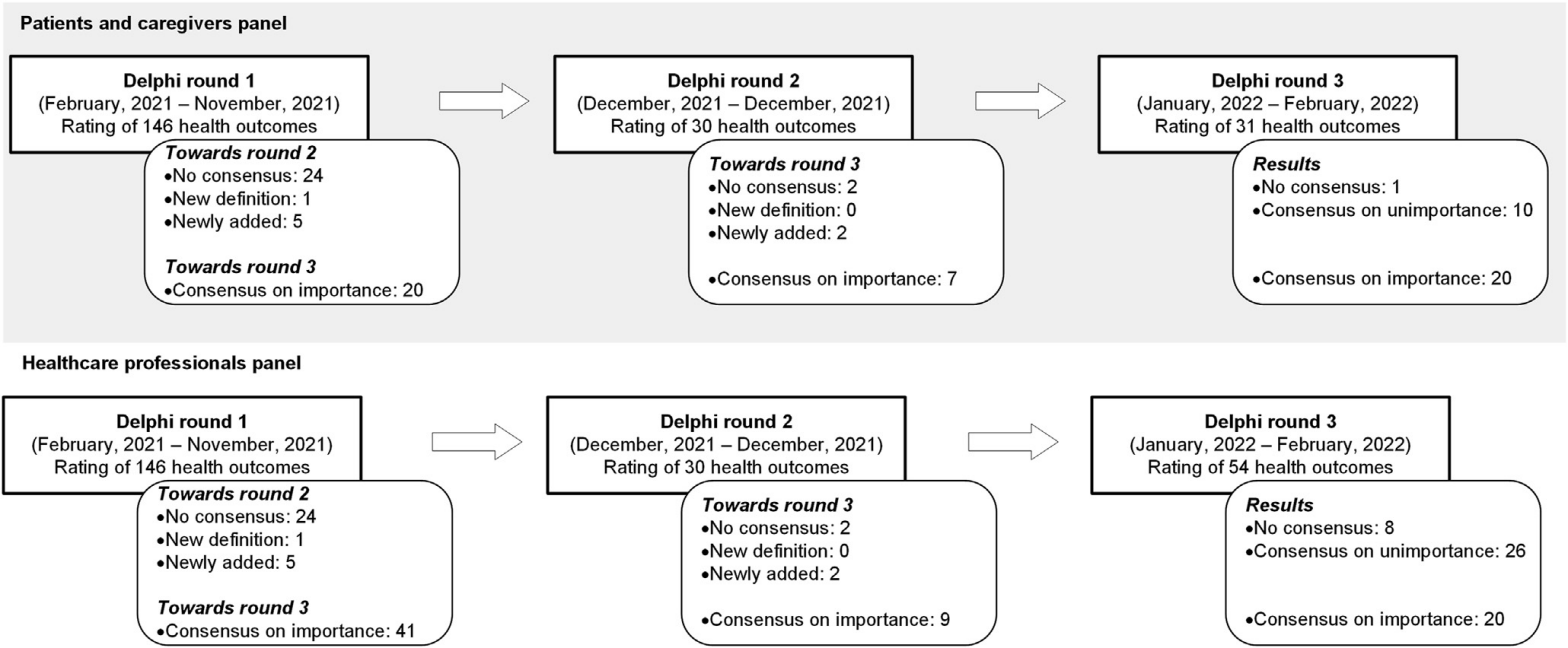
Overview of the Delphi process.

#### 3.2.2 Round 2

In the second round, both panels rated the importance of 30 health outcomes (Supplementary Table S3). The patients and caregivers panel achieved consensus on the importance of 7 health outcomes. The healthcare professionals panel achieved consensus on the importance of 9 health outcomes. Respectively, 21 and 19 health outcomes were excluded from the third round due to consensus on unimportance by the patients and caregivers and healthcare professionals panels. Two new health care outcomes, anxiety and use of on-demand medication, were suggested by the panels.

#### 3.2.3 Round 3

In the third round, the patients and caregivers panel rated the importance of 31 health outcomes whereas the healthcare professionals panel rated 54 health outcomes (Supplementary Table S4). The patients and caregivers, and healthcare professionals panel achieved consensus on unimportance on respectively 10 and 26 health outcomes. The patients and caregivers panel did not achieve consensus on 1 health outcome, whereas the healthcare professionals panel did not achieve consensus on 8 health outcomes.

#### 3.2.4 Outcomes at the end of round 3

At the end of the third Delphi round, both panels individually achieved consensus on the importance of 20 health outcomes. Of these 20 health outcomes, 6 health outcomes are overlapping. In other words, both panels achieved consensus on the importance of 6 health outcomes: 1) number of bleeding episodes per year that require treatment, 2) total number of life-threatening bleeding episodes, 3) severity of the bleeding episode, 4) life-threatening complications, 5) intensity of menstrual bleeding, and 6) impact of menstrual bleeding on daily life (Table 3). Each panel individually achieved consensus on 14 additional health outcomes. The patients and caregivers panel achieved consensus on the importance of health outcomes regarding response to treatment, use of on-demand medication, the impact of menstruation on multiple aspects of daily life, impact of the bleeding disorder on the patients’ emotions, knowledge about the bleeding disorder, and concerns related to the bleeding disorder in general and concerns regarding pregnancy and inheritance of the disease. The healthcare professionals panel achieved consensus on the importance of several health outcomes related to bleeding episodes, treatment side effects including inhibitor development and allergic/hypersensitivity reactions, the presence and degree of joint damage and the interference of pain on the patients’ daily life.

**Table 3:** Health outcomes that are important for patients with autosomal inherited bleeding disorders.

			Consensus achieved by panel(s)	
#	Health outcomes	Definition	Patients and caregivers	Healthcare professionals
***Bleeding episodes***				
10	Frequency of bleeding episodes	The number of bleeding episodes within a year		✓
11	Number of bleeding episodes per year that require treatment	The number of bleeding episodes that require treatment per year	✓	✓
5	Total number of bleeding episodes	The total number of bleeding episodes including severe, life-threatening, and intracranial bleeds		✓
6	Total number of severe bleeding episodes	The total number of severe bleeding episodes a person has experienced		✓
7	Total number of life-threatening bleeding episodes	The total number of life-threatening bleeding episodes the person has experienced	✓	✓
8	Total number of intracranial bleeds	The total number of intracranial bleeds a person has experienced		✓
9	Severity of the bleeding episode	The severity of the bleeding episode	✓	✓
***Treatment***				
12	Response to treatment	How well does a person respond to treatment	✓	
152	Use of on-demand medication	The use of on-demand medication for a person’s bleeding disorder	✓	
48	Treatment side effects	The occurrence of treatment side effects including inhibitor status, allergic reactions		✓
50	Allergic/hypersensitivity reactions	Allergic reactions to treatment		✓
51	Inhibitor development	The presence of antibodies against factor concentrates and/or platelets		✓
52	Inhibitor recurrence	The repeated development of antibodies against factor concentrates and/or platelets		✓
***Joint health***				
22	Joint damage	The presence of joint damage due to joint bleeding episodes		✓
25	Presence of target joints	The presence and number of target joints		✓
26	Alteration in joint functional status	The alteration (improvement/deterioration) in joint functional capacity to perform functions of daily living		✓
151	Increase in joint bleeds	Has the number of joint bleeds increased in a short period?		✓
***Complications***				
57	Life-threatening complications	The occurrence of complications that threaten a person’s life	✓	✓
***Menstruation***				
123	Intensity of menstrual bleeding	Number of times a person needs to change their menstrual products per day	✓	✓
122	Duration and frequency of menstrual bleeding	The duration and frequency of menstrual bleeding		✓
125	Impact of menstruation on daily life	The impact of menstrual bleeding on a person’s ability to engage in activities of daily living (including social relations, work/school)	✓	✓
127	Impact of menstruation on work/school absentee-ism	The impact of menstrual bleeding on a person’s ability to go to work/school	✓	
128	Impact of menstruation on maintaining social relationships	The impact of menstrual bleeding on a person’s ability to maintain social relationships	✓	
129	Impact of menstruation on family life	The impact of menstrual bleeding on a person’s family life	✓	
130	Impact of menstruation on the ability to enjoy life	The impact of menstrual bleeding on a person’s ability to enjoy life	✓	
131	Impact of menstruation on sleep	The impact of menstrual bleeding on a person’s sleep pattern	✓	
***Pain***				
38	Pain interference	The presence of pain and the interference with daily life		✓
***Knowledge, concerns, and impact of having a bleeding disorder***				
137	Knowledge about the bleeding disorder	A person’s level of knowledge about the possible consequences and risks associated with a bleeding disorder	✓	
140	Concerns about the bleeding disorder	A person’s concern about his/her bleeding disorder	✓	
147	Concerns about inheritance of the bleeding disorder	The extent to which a person is concerned about the inheritance of his/her bleeding disorder	✓	
148	Concerns about pregnancy/miscarriage/giving birth	The extent to which a person is concerned about a possible pregnancy/miscarriage or giving birth	✓	
136	Ability to maintain basic self-care	The extent to which a person is able to continue to care for himself/herself as he/she gets older	✓	
110	Impact on emotions	The impact of the bleeding disorder on a person’s emotions	✓	
27	Age at diagnosis	The age at which the bleeding disorder was diagnosed by a hematologist	✓	

## 4 Discussion

In this 3-round Delphi study, we aimed to assess which health outcomes are important for patients with autosomal inherited bleeding disorders as seen from the perspectives of patient, caregiver, and healthcare professional. Our study shows that both the patient and caregivers’ and healthcare professionals’ panel reached consensus on the importance of the following 6 health outcomes for patients with autosomal inherited bleeding disorder, namely: 1) number of bleeding episodes per year that require treatment, 2) total number of life-threatening bleeding episodes, 3) severity of the bleeding episode, 4) life-threatening complications, 5) intensity of menstrual bleeding, and 6) impact of menstrual bleeding on daily life.

In addition to these health outcomes, the patients’ and caregivers’ panel and the healthcare professionals’ panel independently identified several other health outcomes that they deem to be important for patients with autosomal inherited bleeding disorders. Patients and caregivers reached consensus on the importance of health outcomes related to menstruation and the impact of bleeding disorders on their daily lives. In contrast, healthcare professionals found health outcomes related to treatment side effects, joint health, and pain to be of specific importance for patients with autosomal inherited bleeding disorders. These additional health outcomes identified by the different panels highlight the differences among patients’, caregivers’ and healthcare professionals’ perspectives.

The results of this study are comparable to previous studies that aimed to identify a set of important health outcomes for persons with hemophilia. While those studies did not make a distinction between the patient, caregiver, and healthcare professional perspective, several other studies also identified consensus on the importance of the following health outcomes for persons with hemophilia: frequency of bleeding episodes [19], severity of bleeding episodes [12], complications [12], pain [12,13,19,20], joint health [13], and ability to engage in activities of daily life [12,13,20]. None of the studies performed in persons with hemophilia identified the importance of health outcomes related to menstruation. This can be explained by the X-linked inheritance of hemophilia; hemophilia predominantly occurs in males [12]. The considerable resemblance, however, between the other health outcomes identified in this study as important for patients with autosomal inherited bleeding disorders and the outcome sets determined for persons with hemophilia, indicates limited differences between health outcomes found to be important for persons with hemophilia and patients with autosomal inherited bleeding disorders.

### 4.1 Strengths and limitations

To our knowledge, this study is the first to assess which health outcomes are important for patients with autosomal inherited bleeding disorders, as seen from patients’, caregivers’, and healthcare professionals’ perspectives. By choosing to create 2 Delphi panels, one with patients and caregivers and one with healthcare professionals, we were able to distinguish potential differences in the importance of health outcomes across the 2 panels. This study identified that healthcare professionals focus more on disease-related health outcomes that are measurable or influenced by the handling of healthcare professionals, while patients and caregivers focus more on the possible influence of the bleeding disorder on the persons’ emotions and various aspects of their daily lives. This additional insight into the importance of health outcomes from both perspectives provides valuable information to guide the implementation of value-based health care in this patient population.

This study also has several limitations. First, this Delphi study used a long list of 146 health outcomes based on the long list of previous research performed in persons with hemophilia [12,19]. This list was enriched with the findings of a systematic literature review on relevant patient-reported outcomes in patients with autosomal inherited bleeding disorders [14]. In our aim to present the participants with a list of all possibly relevant health outcomes, no critical appraisal was performed on the health outcomes mentioned in the long list of previous studies. Therefore, our study may have included health outcomes that were similar or indistinguishable from each other in the perception of the participants. In addition, some health outcomes included within the long list could not strictly be classified as a health outcome, ie, the health outcome may not represent a change in health status that can be measured clinically, observed, or self-reported [21,22].

Second, within this Delphi study, a very strict definition of importance was used to determine which health outcomes should be included in the second or third round. We defined importance as a median of 5 (very important) and unimportance as a median <5 across all participants within a panel. This strict definition of importance was proven necessary after the first Delphi round. Without this strict definition of importance, it would not have been possible to achieve consensus on a feasible number of important health outcomes within a reasonable number of Delphi rounds. However, this may also have led to the exclusion of health outcomes early on in the Delphi process that, given a different definition of importance and more Delphi rounds, could have been identified as important for patients with autosomal inherited bleeding disorders as seen from the patients’, caregivers’, and healthcare professionals’ perspectives.

Third, we were unable to report race or ethnicity as a social-cultural determinant of health. Therefore, we could not assess its influence on the patients’, caregivers’, and healthcare professionals’ perspectives on the importance of the various health outcomes.

Lastly, difficulties were experienced during the recruitment of patients, caregivers, and healthcare professionals. To obtain a reliable outcome of a Delphi panel, the panel must consist of a minimum of 30 experts [23], in this case, patients, caregivers, and healthcare professionals. To meet these criteria, the first Delphi round was extended multiple times resulting in a first Delphi round that lasted from February 1, 2021 to November 30, 2021. As a result, some participants completed the first Delphi survey in February, while others completed the same survey in November. This delay between the first and second Delphi round may have led to a slightly lower response rate on the second and subsequently third Delphi round. The experienced difficulties during the recruitment of patients, caregivers, and healthcare professionals might also have repercussions for the results of this study. While patients with all types of autosomal inherited bleeding disorders were approached and encouraged to participate in this study, the patients and caregivers panel mainly includes patients with von Willebrand disease. The results of this panel might be less representative for patients with an inherited platelet function disorder or rare bleeding disorder. The rare nature of these diseases makes them inherently difficult to study.

### 4.2 Implications for research and practice

The identification of which health outcomes are important for patients with autosomal inherited bleeding disorders is the first step toward value-based health care implementation in this patient population. Health services aiming to implement value-based health care can use our results to guide their decisions on which health outcomes should be measured in clinical care to assess and improve patient value for patients with inherited bleeding disorders. Additional validation, however, in both a national and international setting is recommended to ensure that the health outcomes identified in this study are also seen as important by a wider group of patients with autosomal inherited bleeding disorders, their caregivers, and healthcare professionals.

To assess patient value in a reliable manner across bleeding disorders, populations, research, and clinical practice, further operationalization of the identified health outcomes is crucial. Subsequently, it is essential to reach consensus on a methodology or set of measurement tools to assess the identified important health outcomes for patients with autosomal inherited bleeding disorders. Future studies are needed to reach consensus on which measurement instruments best capture the value of the identified health outcomes and, in the case of nonexisting disease-specific instruments, to create a suitable, valid, and reliable measurement instrument.

Furthermore, additional research could focus on synthesizing these study results and the various comparable studies performed in persons with hemophilia into one overarching recommended set of important health outcomes for all types of bleeding disorders. This would create uniformity and facilitate the implementation of value-based health care and value assessment by patients with inherited bleeding disorders.

### 4.3 Conclusion

This Delphi consensus study identified which health outcomes are important for patients with autosomal inherited bleeding disorders as seen from the patients’, caregivers’, and healthcare professionals’ perspectives. Both the patients and caregivers panel and the healthcare professionals panel reached consensus on the importance of 6 health outcomes related to bleeding episodes, complications, and menstruation. In addition to these health outcomes, the patients and caregivers panel and the healthcare professionals panel each reached consensus on a different set of important health outcomes, highlighting the differences among the patients’, caregivers’, and healthcare professionals’ perspectives. The identified health outcomes should be considered when implementing value-based health care for patients with autosomal inherited bleeding disorder, although further operationalization is needed.
