# Supplementary material for: Patient-relevant health outcomes for von Willebrand disease, platelet function disorders, and rare bleeding disorders: a Delphi study
Source: Res Pract Thromb Haemost. 2023 Sep 14;7(7):102201. doi: 10.1016/j.rpth.2023.102201 (PMC10579528; doi:10.1016/j.rpth.2023.102201)
Supplement: Supplementary Tables [file mmc1.docx]

**Supplement to:**

**Patient-relevant health outcomes for von Willebrand disease, platelet function disorders and rare bleeding disorders: A Delphi study**

**Running title: Outcomes for autosomal inherited bleeding disorders**

Evelien S. van Hoorn*, MSc, Hester F. Lingsma*, PhD, Marjon H. Cnossen^†^, MD, PhD, Samantha C. Gouw^‡^, MD, PhD, for the SYMPHONY consortium

* Department of Public Health, Erasmus University Medical Center Rotterdam, Rotterdam, The Netherlands

† Department of Pediatric Hematology and Oncology, Sophia Children’s Hospital, Erasmus University Medical Center Rotterdam, Rotterdam, the Netherlands.

‡ Department of Pediatric Hematology, Amsterdam University Medical Centers location University of Amsterdam, Amsterdam, the Netherlands

**Corresponding author:** S.C. Gouw, Department of Pediatric Hematology, Amsterdam University Medical Centers location University of Amsterdam, Meibergdreef 9, Amsterdam, the Netherlands. E-mail: s.c.gouw@amsterdamumc.nl

**Content**

**Response rates per Delphi round3**

Supplementary table 1: Response rate per Delphi round3

**Health outcomes 4**

Supplementary table 2: Long-list of health outcomes for first Delphi round4

Supplementary table 3: List of health outcomes for second Delphi round14

Supplementary table 4: List of health outcomes for third Delphi round16

**Response rate per Delphi round**

Table 1: Response rate per Delphi round

|  | Round 1 | Round 2 | Round 3 |
| --- | --- | --- | --- |
| Number of participants that signed informed consent | 49 | 48* | 48* |
| Number of uncompleted Delphi surveys | 8 | 4 | 2 |
| Number of completed Delphi survey | 34 | 30 | 30 |
| Response rate | 85,7% | 70,8% | 66,6% |

* 1 participant withdrew from the study

**Health outcomes**

Table 2: Long-list of health outcomes for first Delphi round

|  |  |  | **Results of the panel(s)** | |
| --- | --- | --- | --- | --- |
| **#** | **Health outcome** | **Description** | **Patients and caregivers** | **Healthcare professionals** |
| 1 | General health | A person’s state of physical, mental, and social wellbeing | × | × |
| 2 | Vitality | The vitality of the patient | × | × |
| 3 | Number of healthy life years | Expected number of years spent in good health | × | × |
| 4 | Changes in vital signs | The change (improvement/deterioration) in the body’s life-sustaining functions | × | × |
| 5 | Total number of bleeding episodes | The total number of bleeding episodes including severe, life threatening and intracranial bleeds | × | ✓ |
| 6 | Total number of severe bleeding episodes | The total number of severe bleeding episodes a person has experienced | × | ✓ |
| 7 | Total number of life-threatening bleeding episodes | The total number of life-threatening bleeding episodes a person has experienced | ✓ | ✓ |
| 8 | Total number of intracranial bleeds | The total number of intracranial bleeds a person has experienced | × | ✓ |
| 9 | Severity of the bleeding episode | The severity of the bleeding episode | ✓ | ✓ |
| 10 | Frequency of bleeding episodes | The number of bleeding episodes within a year | ✓ | ✓ |
| 11 | Number of bleeding episodes per year that require treatment | The number of bleeding episodes that require treatment per year | ✓ | ✓ |
| 12 | Response to treatment | How well does a person respond to treatment | ✓ | ✓ |
| 13 | Total number of exposure days | The total number of days in which a person is exposed to factor concentrates or platelets | × | × |
| 14 | Frequency of infusions/factor or platelet use or other hemostatic medication | The number of infusions/consumptions of factor concentrates or platelets or other hemostatic medication within a certain time period | × | ✓ |
| 15 | Time until recovery | The duration of recovery after a bleeding episode (the presence of long-term limitations) | × | ✓ |
| 16 | Time until return to physical activities after a bleeding episode | The duration of engagement in similar physical activities prior to the bleeding episode | × | ✓ |
| 17 | Impact of treatment on daily life | The impact of treatment on a person’s daily life | × | ✓ |
| 18 | Impact of an alteration in treatment on daily life | The impact an alteration in treatment (e.g. doses change, change in type of factor concentrates) has on a person’s daily life | × | ✓ |
| 19 | Mobility | The mobility and range of motion of a person | × | × |
| 20 | Number of joint bleeding episodes | The total number of joint bleeding episodes | × | × |
| 21 | Frequency of joint bleeding | The total number of joint bleeding episodes per year | × | × |
| 22 | Joint damage | The presence of joint damage due to joint bleeding episodes | × | ✓ |
| 23 | Number of affected joints | The number of joints that are affected due to joint bleeding episodes | × | ✓ |
| 24 | Alterations of target joints | The changes in appearance/disappearance of target joints, the number of target joints, and the target joint bleeding rate | × | × |
| 25 | Presence of target joints | The presence and number of target joints | × | ✓ |
| 26 | Alteration in joint functional status | The alteration (improvement/deterioration) in joint functional capacity to perform functions of daily living | × | ✓ |
| 27 | Age at diagnosis | The age at which the bleeding disorder was diagnosed by a hematologist | ≠ | ≠ |
| 28 | Time between bleeding onset and administration of treatment | The time between bleeding onset and the administration of factor concentrates and/or platelets | × | ✓ |
| 29 | Time to response | The time between the start of the bleeding episode and the response to treatment | × | × |
| 30 | Time necessary to stop the bleed | The time between the start of the bleeding episode and the stop of bleeding | × | × |
| 31 | Time to return to physical activities | The time between the start of bleeding episode and return to being physically active | × | × |
| 32 | Time to return to work/school | The time between (temporary) cessation of work/school due to the bleeding disorder and return to work/school | × | × |
| 33 | Pain | The duration and intensity of general pain | × | × |
| 34 | Menstrual pain | The duration and intensity of menstrual pain | × | × |
| 35 | Chronic pain | The duration and intensity of persistent/chronic pain | × | × |
| 36 | Acute pain | The duration, intensity and frequency of acute pain | × | × |
| 37 | Joint pain/arthralgia | The duration, intensity and frequency of pain in a specific joint or joints | × | × |
| 38 | Pain interference | The presence of pain and the interference with daily life | × | ✓ |
| 39 | Response to pain | The visible way a person expresses their pain (e.g. through observable expressions such as sighing or crying, and expression of the severity of pain such as resting, protecting themselves, facial expressions and asking for help) | × | × |
| 40 | Frequency of emergency department visits | The number of emergency visits due to bleeding disorder-related events per year | × | × |
| 41 | Frequency of hospital admissions | The number of bleeding disorder-related hospital admissions per year | × | × |
| 42 | Frequency of outpatient visits | The number of bleeding disorder-related outpatient visits per year | ≠ | ≠ |
| 43 | Surgeries | The number of bleeding disorder-related surgeries per year | × | × |
| 44 | Readmissions | The number of readmissions per year | × | × |
| 45 | Need for reoperation/revision | The need for bleeding disorder-related reoperation/revision | × | × |
| 46 | Total length of inpatient stay (days) | The total number of bleeding disorder-related inpatient days per year | × | × |
| 47 | Number of days lost (work/school) | The total number of days lost from work or school because of bleeding disorder-related hospital visits per year | ≠ | ≠ |
| 48 | Treatment side effect | The occurrence of treatment side effects including inhibitor status, allergic reactions | × | ✓ |
| 49 | Pain or discomfort induced by treatment | Interference of treatment-related pain/discomfort on daily life/activities of daily living | × | × |
| 50 | Allergic/ hypersensitivity reactions | Allergic reactions to treatment | ✓ | ✓ |
| 51 | Inhibitor development | The presence of antibodies against factor concentrates and/or platelets | × | ✓ |
| 52 | Inhibitor recurrence | The repeated development of antibodies against factor concentrates and/or platelets | × | ✓ |
| 53 | Duration of immune tolerance | Time during which immune tolerance is achieved (i.e. the inhibitor is eradicated) | × | × |
| 54 | Time needed to achieve complete or partial immune tolerance induction | The time between start of immune tolerance induction and the eradication of the inhibitor | × | × |
| 55 | Recurrences of bleeding episodes because of inadequate treatment | Multiple sequential bleeding episodes due to insufficient administration of factor concentrates and/or platelets | × | × |
| 56 | Discomfort of prophylactic treatment | Perceived discomfort as a result of preventive treatment with factor concentrates (e.g. pain, inflammation, and administration difficulties) | × | × |
| 57 | Life-threatening complications | The occurrence of complications that threaten a person’s life | ✓ | ✓ |
| 58 | Anxiety | The presence of general anxiety by a person with a bleeding disorder | × | × |
| 59 | Anxiety specific to events (e.g. having bleeding episode) | The anxiety of a person about the occurrence of specific bleeding disorder-related events | × | ✓ |
| 60 | Depression | The presence of feelings of dejection, despondency, loss of interest, and loss of energy, lasting longer than two weeks | × | × |
| 61 | Other mental illnesses | The presence of conditions, with the exception of anxiety and depression, that cause severe disturbances in a person’s behavior and thinking | × | × |
| 62 | Chronic inflammation of joints | The presence of persistent/chronic inflammation of one or multiple joints | × | × |
| 63 | Development of bleeding disorder-related comorbidities | The development of one or multiple bleeding disorder-related comorbidities | × | × |
| 64 | Risk of falling | Bleeding disorder-related increased risk of falling | × | × |
| 65 | Risk of bone fracture | Bleeding disorder-related increased risk for bone fracture | × | × |
| 66 | Risk of infections | Bleeding disorder-related increased risk of infections | × | × |
| 67 | Infections (all-cause) | The occurrence of an infection | × | × |
| 68 | Blood infections | The occurrence of blood infection (sepsis) | ≠ | ≠ |
| 69 | Cardiovascular risk | Bleeding disorder-related increased risk of cardiovascular disease | × | × |
| 70 | Need for mobility aids | A person’s dependence on, for example, a crutch, walker or wheelchair to move around | × | × |
| 71 | Ability to participate in working life | The extent to which a person is able to participate in working life | × | × |
| 72 | Securing appropriate employment | A person’s ability to secure employment that is appropriate to his/her condition | × | × |
| 73 | Return to same work | A person’s ability to continue the same work after sick leave | ≠ | ≠ |
| 74 | Change in work productivity | The change in work productivity due to bleeding disorder-related events | × | × |
| 75 | Effect of disease on education/ employment and employment-related issues | The influence of the bleeding disorder on a person’s ability to achieve academic/employment results | × | × |
| 76 | School functioning | The capacity to attend school, to achieve academic results and engage in social relationships by a persons with a bleeding disorder | × | × |
| 77 | Impact on academic achievements | The impact of having a bleeding disorder on a person’s academic achievements | × | × |
| 78 | Time lost from work/school | Time lost from work/school attainment due to bleeding disorder-related events | × | × |
| 79 | Physical health | A person’s ability to perform the basic actions (i.e. mobility, strength, and endurance) that are essential for maintaining independence and carrying out more complex activities | × | × |
| 80 | Ability to engage in physical activities | A person’s ability to engage in physical activities | × | × |
| 81 | Sports participation | A person’s ability to participate in sports | × | × |
| 82 | Confidence in ability to participate in sports | The confidence of a person in his/her ability to participate in sports | × | × |
| 83 | Influence of fear for bleedings on sports participation | The influence of fear with regard to anticipated bleeding episodes on sports participation | × | ✓ |
| 84 | Physical functioning | A person’s ability to perform physical functions and daily routine activities, such as walking, eating, dressing and undressing | × | × |
| 85 | Changes in functional status | The change (improvement/deterioration) in functional capacity to perform daily living functions | × | × |
| 86 | Performing age-specific activities | The extent to which a person is able to perform activities typical for his/her age | × | × |
| 87 | Sexual functioning | The extent of being able to experience sexual pleasures and satisfaction when desired | × | × |
| 88 | Fertility | The extent to which a person is able to have children | ≠ | ≠ |
| 89 | Emotional functioning | The extent to which a person is able to express and regulate his/her emotions | × | × |
| 90 | Mental health | The psychological well-being or absence of mental illnesses | × | × |
| 91 | Self-image | The way a person looks at himself; the thoughts, ideas and judgements that a person has about himself | ≠ | ≠ |
| 92 | Identification as a patient | The extent to which a person identifies him/herself as a patient with a bleeding disorder | ≠ | ≠ |
| 93 | Feeling of normalcy in daily life | The extent to which a person feels normal, does not feel like a patient in daily life | × | × |
| 94 | Autonomy | The degree of autonomy that a person experiences | ≠ | ≠ |
| 95 | Needle fear | The extent to which a person is afraid of needles | ≠ | ≠ |
| 96 | Happiness | The extent to which a person feels happy/satisfaction | × | × |
| 97 | Anger | The extent to which a person is irritable, frustrated and aggressive | × | × |
| 98 | Frustration | The frustration of a person that is directly or indirectly related to having a bleeding disorder | × | × |
| 99 | Sleep | The quality and amount of sleep of a person with a bleeding disorder | × | × |
| 100 | Problems due to sleep disorders | The problems a person experiences due to a disturbed sleep pattern such as reduced alertness, sleepiness and fatigue | × | × |
| 101 | Fatigue | General feeling of being tired | ≠ | ≠ |
| 102 | Ability to participate in social roles and activities | A person’s ability to fulfil his/her role in social settings and to maintain meaningful relationships with partners and family | ≠ | ≠ |
| 103 | Satisfaction with social roles and activities | A person’s satisfaction with being able to perform his/her social roles and activities | ≠ | ≠ |
| 104 | Relationship with peers | The quality of the relationships with friends and other acquaintances | × | × |
| 105 | Company | The perceived availability of persons with whom a person can engage in social activities | × | × |
| 106 | Social isolation | The extent to which a person feels excluded by others | ≠ | ≠ |
| 107 | Emotional support | The extent to which a person can express his concerns and feelings to others | ≠ | ≠ |
| 108 | Practical support | The extent to which a person has access to and accepts help from others, e.g. with the household | ≠ | ≠ |
| 109 | Impact of residency | The impact of the bleeding disorder on a person’s residential location | ≠ | ≠ |
| 110 | Impact on emotions | The impact of the bleeding disorder on a person’s emotions | ≠ | ≠ |
| 111 | Impact on sexual health/intimacy | The impact of the bleeding disorder on a person’s sexual health and intimacy | ≠ | ≠ |
| 112 | Impact on family life | The impact of the bleeding disorder on pursuit of a family life, including but not limited to childbearing, romantic/sexual relationships and marriage | × | × |
| 113 | Impact on fatherhood/motherhood | The impact of the bleeding disorder on the choice whether or not to have children and the number of children | × | × |
| 114 | Disease impact on caregivers and/or partners | The impact of the bleeding disorder on the caregiver and/or partner of a person with a bleeding disorder | × | × |
| 115 | Perceived burden of the bleeding disorder | The burden a person experiences from his/her bleeding disorder | × | ✓ |
| 116 | Perceived family impact | The impact of having a child with a bleeding disorder on family life and the ability to engage in family activities | × | × |
| 117 | Perceived family functioning | The perceived family functioning from the perspective of parents of a child with a bleeding disorder | × | × |
| 118 | Perceived stress of parents | The degree of bleeding disorder-related stress experienced by parents of a child with a bleeding disorder | × | × |
| 119 | Perceived impact on parents’ leisure | The impact of having a child with a bleeding disorder on the time a parent can devote to his/her own needs | × | × |
| 120 | Parental concern | The degree of bleeding disorder-related concern experienced by parents of a child with a bleeding disorder | × | ✓ |
| 121 | Impact on child care | The impact of having a child with a bleeding disorder on the ability to find and maintain child care | × | × |
| 122 | Duration and frequency of menstrual bleeding | The duration and frequency of menstrual bleeding | ✓ | ✓ |
| 123 | Intensity of menstrual bleeding | Number of times a person needs to change themselves per day | ✓ | ✓ |
| 124 | Menstrual anxiety | The presence of menstruation related anxiety by a persons with a bleeding disorder (e.g. fear of leakage) | × | × |
| 125 | Impact of menstruation on daily life | The impact of menstrual bleeding on a person’s ability to engage in activities of daily living (including social relations, work/school) | ✓ | ✓ |
| 126 | Impact of menstruation on mood | The impact of menstrual bleeding on a person’s mood | × | × |
| 127 | Impact of menstruation on work/ school absentee-ism | The impact of menstrual bleeding on a person’s ability to go to work/school | ✓ | ✓ |
| 128 | Impact of menstruation on maintaining social relationships | The impact of menstrual bleeding on a person’s ability to maintain social relationships | ✓ | ✓ |
| 129 | Impact of menstruation of family life | The impact of menstrual bleeding on a person’s family life | ≠ | ≠ |
| 130 | Impact of menstruation on the ability to enjoy life | The impact of menstrual bleeding on a person’s ability to enjoy life | ✓ | ✓ |
| 131 | Impact of menstruation on sleep | The impact of menstrual bleeding on a person’s sleep pattern | ✓ | ✓ |
| 132 | Self-management with the treatment process | The extent to which a person takes responsibility for his/her own behavior and well-being within his/her treatment process | ✓ | ✓ |
| 133 | Self-efficacy | A person’s confidence in his/her own ability to successfully manage his/her bleeding disorder | × | × |
| 134 | Perceived disease control | A person’s perception about his/her control over the disease | ✓ | ✓ |
| 135 | Self-care | The extent to which a person is able to take care of himself/herself | ✓ | ✓ |
| 136 | Ability to maintain basic self-care | The extent to which a persons is able to continue to care for himself/herself as he/she gets older | ✓ | ✓ |
| 137 | Knowledge about the bleeding disorder | A person’s level of knowledge about the possible consequences and risk associated with a bleeding disorder | ✓ | ✓ |
| 138 | Support in obtaining and understanding information | The support a person experiences in obtaining and understanding information about his/her bleeding disorder | ✓ | ✓ |
| 139 | Overall concern | The extent to which a person is concerned in general | × | × |
| 140 | Concerns about the bleeding disorder | A person’s concern about his/her bleeding disorder | ≠ | ≠ |
| 141 | Concerns about financial security | A person’s concern about his/her financial security being affected by his/her bleeding disorder | ≠ | ≠ |
| 142 | Attitude towards the future | A person’s attitude towards his/her future | × | × |
| 143 | Development of comorbidities long-term | The occurrence of other diseases on the long-term | × | × |
| 144 | Deterioration of venous access | Deterioration of venous access due to too many infusions at the same site | ≠ | ≠ |
| 145 | Loss of mobility due to prosthesis | The loss of mobility due to insufficient alignment of a prosthesis | ≠ | ≠ |
| 146 | Mortality | The number of bleeding disorder-related deaths per year | × | × |

✓ The panel achieved consensus on importance; × the panel achieved consensus on unimportance; ≠ no consensus was achieved by the panel

Table 3: List of health outcomes for second Delphi round

|  |  |  | **Results of the panel(s)** | |
| --- | --- | --- | --- | --- |
| **#** | **Health outcome** | **Description** | **Patients and caregivers** | **Healthcare professionals** |
| 2 | Vitality* | The extent to which a person feels powerful and energetic, i.e. a person’s vitality | × | × |
| 27 | Age at diagnosis | The age at which the bleeding disorder was diagnosed by a hematologist | ✓ | ✓ |
| 42 | Frequency of outpatient visits | The number of bleeding disorder-related outpatient visits per year | × | × |
| 47 | Number of days lost (work/school) | The total number of days lost from work or school because of bleeding disorder-related hospital visits per year | ≠ | ≠ |
| 68 | Blood infections | The occurrence of blood infection (sepsis) | × | × |
| 73 | Return to same work | A person’s ability to continue the same work after sick leave | × | × |
| 88 | Fertility | The extent to which a person is able to have children | × | × |
| 91 | Self-image | The way a person looks at himself; the thoughts, ideas and judgements that a person has about himself | × | × |
| 92 | Identification as a patient | The extent to which a person identifies him/herself as a patient with a bleeding disorder | × | × |
| 94 | Autonomy | The degree of autonomy that a person experiences | × | ✓ |
| 95 | Needle fear | The extent to which a person is afraid of needles | × | × |
| 101 | Fatigue | General feeling of being tired | ✓ | ✓ |
| 102 | Ability to participate in social roles and activities | A person’s ability to fulfil his/her role in social settings and to maintain meaningful relationships with partners and family | × | × |
| 103 | Satisfaction with social roles and activities | A person’s satisfaction with being able to perform his/her social roles and activities | × | × |
| 106 | Social isolation | The extent to which a person feels excluded by others | × | × |
| 107 | Emotional support | The extent to which a person can express his concerns and feelings to others | × | × |
| 108 | Practical support | The extent to which a person has access to and accepts help from others, e.g. with the household | × | × |
| 109 | Impact of residency | The impact of the bleeding disorder on a person’s residential location | ≠ | ≠ |
| 110 | Impact on emotions | The impact of the bleeding disorder on a person’s emotions | ✓ | ✓ |
| 111 | Impact on sexual health/intimacy | The impact of the bleeding disorder on a person’s sexual health and intimacy | × | × |
| 129 | Impact of menstruation of family life | The impact of menstrual bleeding on a person’s family life | ✓ | ✓ |
| 140 | Concerns about the bleeding disorder | A person’s concern about his/her bleeding disorder | ✓ | ✓ |
| 141 | Concerns about financial security | A person’s concern about his/her financial security being affected by his/her bleeding disorder | × | × |
| 144 | Deterioration of venous access | Deterioration of venous access due to too many infusions at the same site | × | × |
| 145 | Loss of mobility due to prosthesis | The loss of mobility due to insufficient alignment of a prosthesis | × | × |
| 147 | Concerns about inheritance of the bleeding disorder ^†^ | The extent to which a person is concerned about the inheritance of his/her bleeding disorder | ✓ | ✓ |
| 148 | Concerns about pregnancy/miscarriage/giving birth ^†^ | The extent to which a person is concerned about a possible pregnancy/miscarriage or giving birth | ✓ | ✓ |
| 149 | Visualization of the joints (MRI or X-ray) ^†^ | The extent to which the joints have been mapped by MRI or X-ray and how long ago this was done | × | × |
| 150 | Visualization of joint bleeds ^†^ | The extent to which the joint bleeding has been visualized with ultrasound at control moments | × | × |
| 151 | Increase in joint bleeding ^†^ | Has the number of joint bleeds increased in a short period of time? | × | ✓ |

✓ The panel achieved consensus on importance; × the panel achieved consensus on unimportance; ≠ no consensus was achieved by the panel.

* The definition of these health outcomes was changed during the first round.

† These health outcomes were added during the first Delphi round.

Table 4: List of health outcomes for third Delphi round

|  |  |  | **Results of the panel(s)** | |
| --- | --- | --- | --- | --- |
| **#** | **Health outcome** | **Description** | **Patients and caregivers** | **Healthcare professionals** |
| 5 | Total number of bleeding episodes | The total number of bleeding episodes including severe, life threatening and intracranial bleeds |  | ✓ |
| 6 | Total number of severe bleeding episodes | The total number of severe bleeding episodes a person has experienced |  | ✓ |
| **7** | **Total number of life-threatening bleeding episodes** | **The total number of life-threatening bleeding episodes a person has experienced** | ✓ | ✓ |
| 8 | Total number of intracranial bleeds | The total number of intracranial bleeds a person has experienced |  | ✓ |
| **9** | **Severity of the bleeding episode** | **The severity of the bleeding episode** | ✓ | ✓ |
| **10** | **Frequency of bleeding episodes** | **The number of bleeding episodes within a year** | × | ✓ |
| **11** | **Number of bleeding episodes per year that require treatment** | **The number of bleeding episodes that require treatment per year** | ✓ | ✓ |
| **12** | **Response to treatment** | **How well does a person respond to treatment** | ✓ | × |
| 14 | Frequency of infusions/factor or platelet use or other hemostatic medication | The number of infusions/consumptions of factor concentrates or platelets or other hemostatic medication within a certain time period. |  | × |
| 15 | Time until recovery | The duration of recovery after a bleeding episode (the presence of long-term limitations) |  | × |
| 16 | Time until return to physical activities after a bleeding episode | The duration of engagement in similar physical activities prior to the bleeding episode |  | × |
| 17 | Impact of treatment on daily life | The impact of treatment on a person’s daily life |  | × |
| 18 | Impact of an alteration in treatment on daily life | The impact an alteration in treatment (e.g. doses change, change in type of factor concentrates) has on a person’s daily life |  | × |
| 22 | Joint damage | The presence of joint damage due to joint bleeding episodes |  | ✓ |
| 23 | Number of affected joints | The number of joints that are affected due to joint bleeding episodes |  | × |
| 25 | Presence of target joints | The presence and number of target joints |  | ✓ |
| 26 | Alteration in joint functional status | The alteration (improvement/deterioration) in joint functional capacity to perform functions of daily living |  | ✓ |
| **27** | **Age at diagnosis** | **The age at which the bleeding disorder was diagnosed by a hematologist** | ✓ | × |
| 28 | Time between bleeding onset and administration of treatment | The time between bleeding onset and the administration of factor concentrates and/or platelets |  | ≠ |
| 38 | Pain interference | The presence of pain and the interference with daily life |  | ✓ |
| **47** | **Number of days lost (work/school)** | **The total number of days lost from work or school because of bleeding disorder-related hospital visits per year** | × | ≠ |
| 48 | Treatment side effect | The occurrence of treatment side effects including inhibitor status, allergic reactions |  | ✓ |
| **50** | **Allergic/ hypersensitivity reactions** | **Allergic reactions to treatment** | × | ✓ |
| 51 | Inhibitor development | The presence of antibodies against factor concentrates and/or platelets |  | ✓ |
| 52 | Inhibitor recurrence | The repeated development of antibodies against factor concentrates and/or platelets |  | ✓ |
| **57** | **Life-threatening complications** | **The occurrence of complications that threaten a person’s life** | ✓ | ✓ |
| **58** | **Anxiety ^*^** | **The presence of general anxiety by a person with a bleeding disorder** | × | × |
| 59 | Anxiety specific to events (e.g. having bleeding episode) | The anxiety of a person about the occurrence of specific bleeding disorder-related events |  | × |
| 83 | Influence of fear for bleedings on sports participation | The influence of fear with regard to anticipated bleeding episodes on sports participation |  | × |
| 94 | Autonomy | The degree of autonomy that a person experiences |  | × |
| **101** | **Fatigue** | **General feeling of being tired** | × | × |
| **109** | **Impact of residency** | **The impact of the bleeding disorder on a person’s residential location** | ≠ | × |
| **110** | **Impact on emotions** | **The impact of the bleeding disorder on a person’s emotions** | ✓ | × |
| 115 | Perceived burden of the bleeding disorder | The burden a person experiences from his/her bleeding disorder |  | × |
| 120 | Parental concern | The degree of bleeding disorder-related concern experienced by parents of a child with a bleeding disorder |  | ≠ |
| **122** | **Duration and frequency of menstrual bleeding** | **The duration and frequency of menstrual bleeding** | × | ✓ |
| **123** | **Intensity of menstrual bleeding** | **Number of times a person needs to change themselves per day** | ✓ | ✓ |
| **125** | **Impact of menstruation on daily life** | **The impact of menstrual bleeding on a person’s ability to engage in activities of daily living (including social relations, work/school)** | ✓ | ✓ |
| **127** | **Impact of menstruation on work/ school absentee-ism** | **The impact of menstrual bleeding on a person’s ability to go to work/school** | ✓ | ≠ |
| **128** | **Impact of menstruation on maintaining social relationships** | **The impact of menstrual bleeding on a person’s ability to maintain social relationships** | ✓ | ≠ |
| **129** | **Impact of menstruation of family life** | **The impact of menstrual bleeding on a person’s family life** | ✓ | ≠ |
| **130** | **Impact of menstruation on the ability to enjoy life** | **The impact of menstrual bleeding on a person’s ability to enjoy life** | ✓ | ≠ |
| **131** | **Impact of menstruation on sleep** | **The impact of menstrual bleeding on a person’s sleep pattern** | ✓ | ≠ |
| **132** | **Self-management with the treatment process** | **The extent to which a person takes responsibility for his/her own behavior and well-being within his/her treatment process** | × | × |
| **134** | **Perceived disease control** | **A person’s perception about his/her control over the disease** | × | × |
| **135** | **Self-care** | **The extent to which a person is able to take care of himself/herself** | × | × |
| **136** | **Ability to maintain basic self-care** | **The extent to which a persons is able to continue to care for himself/herself as he/she gets older** | ✓ | × |
| **137** | **Knowledge about the bleeding disorder** | **A person’s level of knowledge about the possible consequences and risk associated with a bleeding disorder** | ✓ | × |
| **138** | **Support in obtaining and understanding information** | **The support a person experiences in obtaining and understanding information about his/her bleeding disorder** | × | × |
| **140** | **Concerns about the bleeding disorder** | **A person’s concern about his/her bleeding disorder** | ✓ | × |
| **147** | **Concerns about inheritance of the bleeding disorder** | **The extent to which a person is concerned about the inheritance of his/her bleeding disorder** | ✓ | × |
| **148** | **Concerns about pregnancy/miscarriage/giving birth** | **The extent to which a person is concerned about a possible pregnancy/miscarriage or giving birth** | ✓ | × |
| 151 | Increase in joint bleeds | Has the number of joint bleeds increased in a short period of time? |  | ✓ |
| **152** | **Use of on-demand medication ^*^** | **The use of on-demand medication for a person’s bleeding disorder** | ✓ | × |

All health outcomes in this table were rated by the healthcare professionals panel during the third Delphi round. The health outcomes in **bold** were rated by the patients and caregivers panel.

✓ The panel achieved consensus on importance; × the panel achieved consensus on unimportance; ≠ no consensus was achieved by the panel.

† These health outcomes were added during the second Delphi round. Number 58 was first excluded from the second Delphi round since there was consensus on unimportance.
